# Clinical implication of subcategorizing T2 category into T2a and T2b in TNM staging of breast cancer

**DOI:** 10.1002/cam4.1831

**Published:** 2018-10-12

**Authors:** Jiwoong Jung, Young Jin Suh, Byung Kyun Ko, Eun Sook Lee, Eun‐Kyu Kim, Nam Sun Paik, Kyung Do Byun, Ki‐Tae Hwang, Sei Hyun Ahn, Dong‐Young Noh, Seok Jin Nam, Byeong‐Woo Park, Woo Chul Noh, Jung Han Yoon, Soo Jung Lee, Eun Kyu Lee, Joon Jeong, Sehwan Han, Ho Yong Park, Nam‐Sun Paik, Young Tae Bae, Hyouk Jin Lee, Heung kyu Park, Seung Sang Ko, Woo‐Chan Park, Sung Hoo Jung, Se Heon Cho, Sei Joong Kim, Se Jeong Oh, Ku Sang Kim, Chanheun Park, Byung Joo Song, Je Ryong Kim, Jeoung Won Bae, Jeong‐Soo Kim, Sun Hee Kang, Geumhee Gwak, Jee Hyun Lee, Tae Hyun Kim, Myungchul Chang, Sung Yong Kim, Jung Sun Lee, Jeong‐Yoon Song, Hai Lin Park, Sun Young Min, Jung‐Hyun Yang, Sung Hwan Park, Jong‐Min Baek, Lee Su Kim, Dong Won Ryu, Kweon Cheon Kim, Min Sung Chung, Hee Boong Park, Cheol Wan Lim, Un Jong Choi, Beom Seok Kwak, Young Sam Park, Hyuk Jai Shin, Young Jin Choi, Doyil Kim, Airi Han, Jong Hyun Koh, Sangyong Choi, Daesung Yoon, Soo Youn Choi, Shin Hee Chul, Jae Il Kim, Jae Hyuck Choi, Jin Woo Ryu, Chang Dae Ko, Il Kyun Lee, Dong Seok Lee, Seunghye Choi, Youn Ki Min, Young San Jeon, Eun‐Hwa Park

**Affiliations:** ^1^ Department of Surgery Seoul Medical Center Seoul Korea; ^2^ Department of Surgery St. Vincent's Hospital College of Medicine The Catholic University of Korea Suwon Korea; ^3^ Department of Surgery Ulsan University Hospital University of Ulsan College of Medicine Ulsan Korea; ^4^ Center for Breast Cancer Research Institute and Hospital National Cancer Center Goyang Korea; ^5^ Department of Surgery Seoul National University Bundang Hospital Seoul National University College of Medicine Seongnam Korea; ^6^ Department of Surgery Ewha Womans University Mokdong Hospital Ewha Womans University College of Medicine Seoul Korea; ^7^ Department of Surgery Dong‐A University Medical Center Dong‐A University College of Medicine Busan Korea; ^8^ Department of Surgery Seoul National University Boramae Medical Center Seoul Korea

**Keywords:** breast neoplasms, prognosis, TNM staging, tumor staging

## Abstract

Regarding TNM staging in breast cancer, T2 category is currently not divided into subcategories even though it covers a wider range of tumor sizes than T1 category. Using Korean Breast Cancer Registry database, data of 41 071 women diagnosed as non‐metastatic T2 breast cancer between 2001 and 2014 were analyzed. Cutoff value for optimal tumor size was approximated by receiver operating characteristic (ROC) curve to subcategorize T2 tumors. Overall survival (OS) was compared between two subcategories. Median follow‐up period was 65 months. Of 41 071 patients, 4504 (11.0%) died. Based on ROC curve analysis, 3.0 cm was selected as the cutoff value. Five‐year OS rate was 91% in patients with breast tumors ≤3.0 cm (T2a) and 86% in patients with breast tumors >3.0 cm (T2b) (log‐rank *P *<* *0.001). T2b subcategory showed worse OS than T2a subcategory regardless of node status (log‐rank *P *<* *0.001 for all node categories). Within every subgroup defined by primary OS analysis covariates, T2b subcategory consistently showed worse outcome compared to T2a subcategory. By multivariate analysis, T2b subcategory was a significant independent prognostic factor of OS (hazard ratio: 1.26, 95% CI = 1.18‐1.34). T2 category of breast cancer could be subcategorized into T2a and T2b with a cutoff value of 3 cm. These subcategories definitely showed different OSs even after adjusted for known prognostic factors. Subcategorization of T2 category might be useful for predicting prognosis more accurately and tailoring adjuvant therapy.

## 1 INTRODUCTION

With various adjuvant therapies for breast cancer, accurate estimate of outcomes is becoming increasingly important for appropriate tailored therapy. Currently, several prognostic factors have been identified, including tumor size, regional lymph node (LN) involvement, the presence of distant metastasis, age, histologic grade, the presence of lymphovascular invasion, hormone receptor, and human epidermal growth factor receptor 2 (HER2) status. Among these risk factors, tumor size along with regional LN status and the presence of distant metastasis constitute the basis of American Joint Committee on Cancer (AJCC) staging system.^1^ Despite recent emergence of new prognostic factors and gene expression profiling,^2^, ^3^, ^4^ tumor size remains a key factor in cancer biology. It is used in all guidelines for breast cancer and prognosis prediction models, such as Adjuvant! Online.^5^

Traditionally, AJCC cancer staging manual for breast cancer determines T category with two cutoff values (2.0 and 5.0 cm) in the longest diameter. However, the prognostic value of this categorization has been questioned for triple‐negative or basal‐like subtypes while it is overrepresented in other subtypes.^6^, ^7^ A recent prospective study involving women with breast cancer has shown that prognoses of those whose tumors have favorable molecular features are similar regardless of whether their tumors are measured greater than or less than 2 cm.^8^ On the other hand, Yu et al^9^ have reported that larger tumor size (greater than 6.0 cm) with negative LNs might be a surrogate for biologically indolent disease of distant dissemination. Results of these studies combined indicate that current classification of tumor size might be too simplistic to categorize heterogeneous breast cancer, especially for larger sized tumors.

In this context, recent clinical trials for early breast cancer patients not only included T1 breast cancers, but also included T2 diseases as inclusion criteria for tumor size.^8^, ^10^ Some may think that biologic characteristics of a tumor are more relevant to breast cancer prognosis than the size of the tumor. However, current classification of tumor size in breast cancers is insufficient to predict accurate prognosis or intrinsic tumor biology. Current edition of AJCC cancer staging manual subcategorizes T1 breast cancers (tumor size measures 2 cm or less) into several subcategories to help guide treatment since its 3rd edition.^1^ Many clinical studies regarding small breast cancers are based on this subcategorization of T1 breast cancers. Those studies have given us better knowledge of small breast cancers. T2 category is currently not divided into subcategories even though it covers a wider range of tumor sizes than T1 category. Since heterogeneity of breast neoplasm is not confined to small breast cancer, subcategorization of T category for tumors larger than 2.0 cm might be needed. The objective of this study was to assess the feasibility of T2 subcategorization according to tumor size and determine the prognostic impact of such subcategorization using a large database for analysis.

## 2 METHODS

### 2.1 KBCR database

The Korean Breast Cancer Society (KBCS) started collecting national data on patients with breast cancer since 1996. Korean Breast Cancer Registry (KBCR) is a prospectively maintained database of KBCS. Nationwide, breast surgeons in 102 teaching hospitals participate in this program. KBCR started offline in 1996. It has been converted to an online registration project since 2001. This registry included about 35% of all newly diagnosed breast cancer patients in Korea before 2001. After online conversion, registration rate has been increased to about 50%.^11^, ^12^, ^13^, ^14^ From 1996 through 2015, more than 160 000 breast cancer patients were registered to this registry. Detailed information regarding the KBCR database has been reported previously.^15^

### 2.2 Patients selection and follow‐up

A total of 53 878 pathologic T2 breast cancer patients were selected from KBCR, including 162 520 breast cancer patients as of 2015. For our analysis, we excluded 842 patients with distant metastases. Subsequently, 323 patients with malignant phyllodes tumors or lymphomas and 115 patients diagnosed before age of 18 years were also excluded. Additionally, 2168 patients who received neoadjuvant chemotherapy or hormonal therapy were excluded. Data for the remaining 50 430 patients were included for primary analysis. KBCR data do not include information about tumor recurrence because Korean Central Cancer Registry and Statistics Korea only provide mortality data. Total overall survival data were updated from Statistics Korea until December 31, 2014 (http://kosis.kr).^16^ Data of 1664 patients diagnosed after 2014 were excluded from this study. Additionally, data of 7695 patients diagnosed before 2001, the year of starting online registration, were excluded because the registration rate was relatively lower than that after online registration. Especially, using data before 1996 could cause possible selection bias because those data were registered by retrospective review. After exclusion, data for the remaining 41 071 patients were used for final analyses. The selection process of the study cohort is summarized in Figure S1.

### 2.3 Tumor size

During online data input process of KBCR, T category was one of required entries at the first page of online registration. Tumor size of breast cancer was an optional entry in the registration process and occasionally omitted. For that reason, we used T category as major inclusion criteria of this study. It was regarded as a more reliable variable than input value of tumor size. Cases without information about tumor size were included in primary analysis of baseline characteristics. They were excluded for subsequent analyses to subcategorize T2 category.

### 2.4 Statistical analysis

Overall survival (OS) was defined as the time from the first diagnosis of primary breast cancer to death from any cause. Survival curves were estimated by the Kaplan‐Meier method. Log‐rank tests were used for comparison of survival curves. Time point survival was estimated using life table method. Cox's proportional hazard regression model was used to calculate adjusted hazard ratios (HRs) with 95% confidence intervals (CIs). Chi‐square test was used to determine differences in clinicopathologic features between pairs of groups. Receiver operator characteristic (ROC) curves were used to determine the optimal cutoff value of tumor size. All statistical analyses were carried out using SPSS, version 20.0 (SPSS Inc., Chicago, IL, USA). Two‐sided *P *<* *0.05 was considered statistically significant.

## 3 RESULTS

### 3.1 Baseline characteristics of study population

Of 41 071 patients analyzed, 34 528 (84.1%) had data of tumor sizes for analyses. Of these 34 528 patients, more than two‐thirds had breast tumors measuring between 2.0 and 3.0 cm. Patients who had tumors measuring between 3.0 and 5.0 cm were less than one‐third of them. More than half of these patients had no regional LN involvement. Among them, 58.4% were estrogen receptor (ER) or progesterone receptor (PR) positive while 18.0% had HER2 gene overexpression. Median follow‐up period was 65 months. Of 41 071 patients, 4504 (11.0%) died. Baseline characteristics and tumor size distributions of these patients are summarized in Table 1.

**Table 1:** Baseline characteristics of patients with T2 tumors in KBCR

Patient characteristics	Total (N = 41 071)		Tumor size ≤3.0 cm (n = 23 535)		Tumor size >3.0 cm (n = 10 993)		*P*
	No.	%	No.	%	No.	%	
Type of operation							
BCS	18 471	45.0%	11 771	50.0%	3414	31.1%	<0.001
Mastectomy	22 137	53.9%	11 531	49.0%	7473	68.0%	
etc.	463	1.1%	233	1.0%	106	0.9%	
Year of operation							
2001‐2005	11 940	29.1%	6606	28.1%	3175	28.9%	<0.001
2006‐2010	15 723	38.3%	9182	39.0%	4685	42.6%	
2011‐2014	13 408	32.6%	7747	32.9%	3133	28.5%	
Patient age, years							
≤50	24 064	58.6%	13 683	58.1%	6591	60.0%	0.001
>50	17 007	41.4%	9852	41.9%	4402	40.0%	
Tumor size							
>2.0 and ≤2.5	15 280	37.2%					
>2.5 and ≤3.0	8255	20.1%					
>3.0 and ≤3.5	4996	12.2%					
>3.5 and ≤4.0	3182	7.7%					
>4.0 and ≤4.5	1687	4.1%					
>4.5 and ≤5.0	1128	2.7%					
Unknown	6543	15.9%					
N category							
N0	20 969	51.1%	12 747	54.2%	4682	42.6%	<0.001
N1	12 980	31.6%	7192	30.6%	3458	31.5%	
N2	4479	10.9%	2305	9.8%	1674	15.2%	
N3	2533	6.2%	1234	5.2%	1156	10.5%	
Unknown	110	0.3%	57	0.2%	23	0.2%	
Histologic grade							
1	3284	8.0%	2226	9.5%	811	7.4%	<0.001
2	14 262	34.7%	9458	40.2%	3910	35.6%	
3	16 068	39.1%	9924	42.2%	5301	48.2%	
Unknown	7457	18.2%	1927	8.1%	971	8.8%	
Lymphovascular invasion							
Yes	13 392	32.6%	8343	35.4%	4435	40.3%	<0.001
No	15 103	36.8%	10 505	44.6%	3779	34.4%	
Unknown	12 576	30.6%	4687	19.9%	2779	25.3%	
ER status							
Positive	22 365	54.5%	14 535	61.8%	6388	58.1%	<0.001
Negative	13 737	33.4%	8445	35.9%	4355	39.6%	
Unknown	4969	12.1%	555	2.4%	250	2.3%	
PR status							
Positive	19 842	48.3%	12 930	54.9%	5620	51.1%	<0.001
Negative	16 088	39.2%	10 049	42.7%	5112	46.5%	
Unknown	5141	12.5%	556	2.4%	261	2.4%	
HER2 overexpression							
Positive	7403	18.0%	4699	20.0%	2274	20.7%	<0.001
Negative	23 017	56.0%	15 036	63.9%	6482	59.0%	
Unknown	10 651	25.9%	3800	16.1%	2237	20.3%	
Chemotherapy							
Yes	30 202	73.5%	19 117	81.2%	9033	82.2%	<0.001
No	3787	9.2%	2471	10.5%	924	8.4%	
Unknown	7082	17.2%	1944	8.3%	1036	9.4%	
Radiation therapy							
Yes	18 503	45.1%	12 301	52.3%	4903	44.6%	<0.001
No	13 563	33.0%	8243	35.0%	4350	39.6%	
Unknown	9005	21.9%	2991	12.7%	1740	15.8%	
Endocrine therapy							
Yes	20 126	49.0%	13 718	56.0%	5706	51.9%	<0.001
No	10 630	25.9%	6613	28.1%	3224	29.3%	
Unknown	10 315	25.1%	3744	15.9%	2063	18.8%	
Median follow‐up, months (IQR)	65 (31‐105)	65 (32‐105)	68 (33‐103)				
Death, all causes	4504	11.0%	2225	9.5%	1590	14.5%	

BCS, breast‐conserving surgery; ER, estrogen receptor; HER2, human epidermal growth factor receptor 2; IQR, interquartile range; KBCR, Korean Breast Cancer Registry; PR, progesterone receptor.

### 3.2 Determination of optimal cutoff value

**Figure 1:**
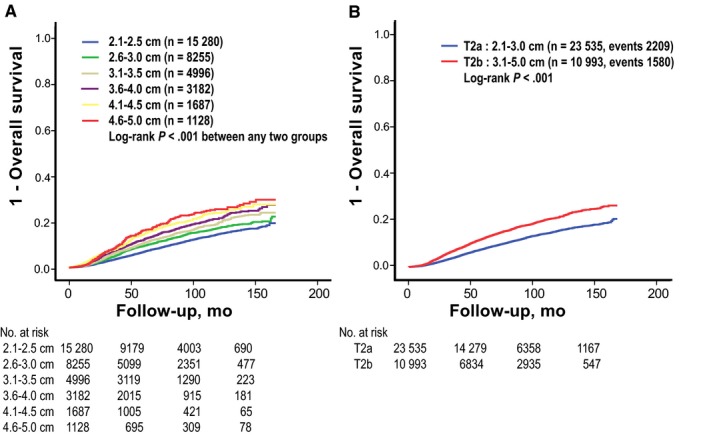
Survival curves for T2 breast cancer patients stratified by categorical tumor size. A, Six groups categorized by interval of 0.5 cm; B, Two T2 subcategories divided by a cutoff value of 3.0 cm (tentatively named T2a and T2b). OS, overall survival

First, we categorized the study population into six groups according to tumor size with an interval of 0.5 cm. When survival outcomes of these six groups were compared, OS was negatively correlated with tumor size (Figure 1A). Between any two groups among these six T2 subgroups, OS differed from each other. To determinate an optimal cutoff of tumor size, we examined ROC curves from tumor size and patients’ survival outcome. Optimal cutoff point subdividing T2 category into two subcategories was found to be 2.95 cm in this study population (Figure S2). We selected 3.0 cm instead of 2.95 cm as a cutoff value because of its simplicity as a representative value.

### 3.3 Comparison of two T2 subcategories

Of 34 528 patients analyzed, 23 535 (68.2%) had tumors with size between 2.0 and 3.0 cm (tentatively named T2a) while 10 993 (31.8%) patients had tumors with size between 3.0 and 5.0 cm (tentatively named T2b). Patient and tumor characteristics of each subcategory are presented in Table 1. Patients with T2b breast tumors showed more advanced node categories than patients with T2a (*P *<* *0.001). T2b tumors also showed different features from T2a tumors in histologic grade, hormone receptor status, HER2 gene expression status, and lymphovascular invasiveness. The ratio of T2a to T2b did not change until 2010. After that, the proportion of T2a disease was increased (*P *<* *0.001). Patients with T2b tumors showed worse OS than patients with T2a tumors (HR: 1.51; 95% CI = 1.42‐1.61; log‐rank *P *<* *0.001; Figure 1B). Five‐year OS rate was 91% in T2a patients and 86% in T2b patients.

### 3.4 Prognostic significance of T2 subcategorization

**Figure 2:**
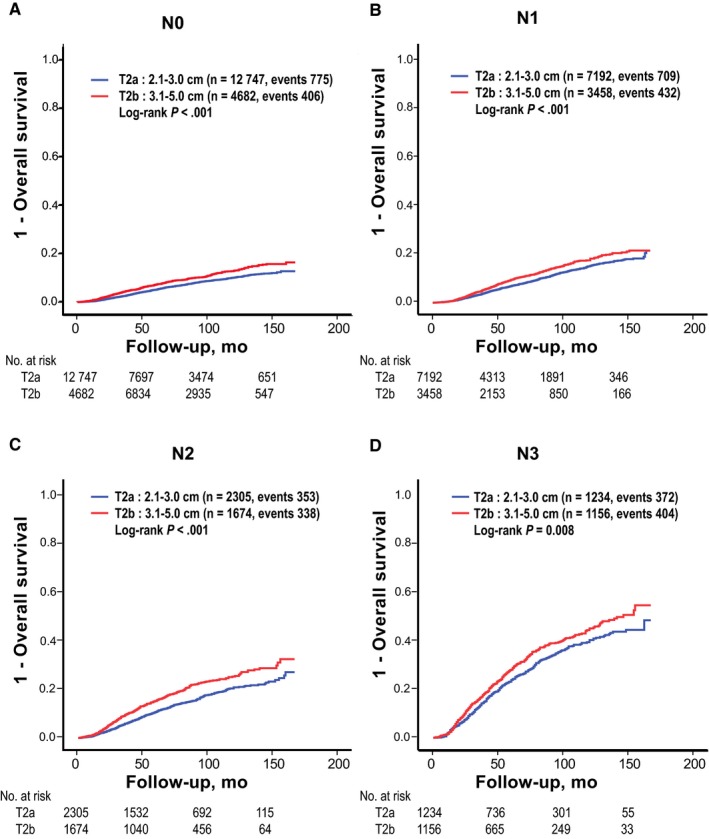
Survival comparison between two T2 subcategories within each node category. A, N0; B, N1; C, N2; and D, N3. Women with T2b breast cancers showed consistently worse outcomes in every node category than women with T2a breast cancers

**Figure 3:**
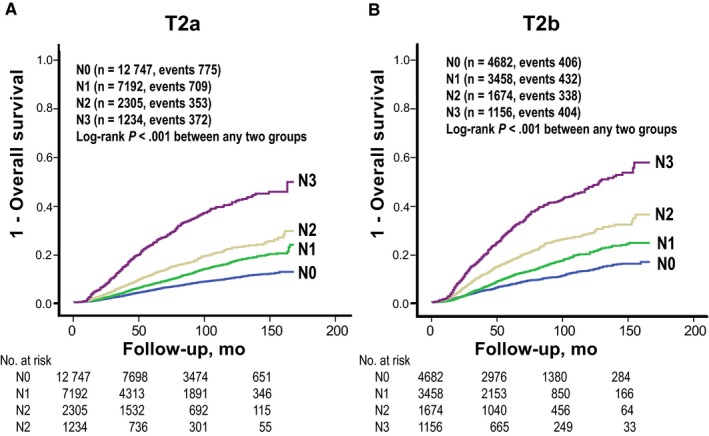
Survival curves for each node category within two T2 subcategories. A, T2a, 2.1‐3.0 cm and B, T2b, 3.1‐5.0 cm. Advanced node categories similarly worsened the prognosis within any T2 subcategory

To determinate influences of different node status on the two T2 subcategories, the effect of T2 subcategorization was evaluated for each pathologic node category. Patients with T2b breast cancer showed consistently worse survival outcome than patients with T2a regardless of pathologic node categories (log‐rank *P *<* *0.001 in all node categories; Figure 2). Survival differences between T2a and T2b were more pronounced in advanced node categories (N2 and N3) than those in early node category (N0 and N1). Additionally, within each T2 subcategory, prognostic values of pathologic node categories were reevaluated. Although distributions of node categories were quite different between the two T2 subcategories, pathologic node category was still a powerful prognostic factor in both subcategories. Within any T2 subcategory, the presence of positive lymph node was associated with worse prognosis (Figure 3). These findings suggest that the prognostic value of T2 subcategorization is independent of pathologic node categories. We then performed subgroup analyses according to breast operation type, ER status, PR status, HER2 gene expression status, histologic grade, nuclear grade, lymphovascular invasion, receipt of adjuvant therapy, and age (50 years or more vs less than 50 years). In every subgroup, T2b breast cancer patients consistently showed worse outcome than T2a breast cancer patients (Table 2). In univariate survival analyses, long‐term outcomes were significantly different according to pathologic node categories, ER status, PR status, HER2 gene expression status, histologic or nuclear grade, lymphovascular invasion, age (50 years or more vs less than 50 years), receipt of adjuvant therapy, and T2 subcategory. T2b subcategory (tumors > 3 cm) was a significant independent risk factor affecting OS in a model with variables including tumor size (≤ 3 cm vs > 3 cm), LN status (N0 vs N1 vs N2 vs N3), histologic grade (grade 1 vs 2 vs 3), lymphovascular invasion (negative vs positive), ER and PR status (negative vs positive), HER2 gene overexpression status (negative vs positive), receipt of adjuvant chemotherapy (no vs yes), receipt of adjuvant radiation therapy (no vs yes), receipt of endocrine therapy (no vs yes), age (less than 50 years vs 50 years or more), and time of operation (2001‐2005 vs 2006‐2010 vs 2011‐2014). In multivariate analyses, patients with breast cancer size larger than 3.0 cm had a HR for OS of 1.26 (95% CI = 1.18‐1.34). Advanced node categories, higher histologic grade, ER negativity, PR negativity, omission of any adjuvant therapy, older age (50 years or more), and past year of operation were also independent risk factors in multivariate analyses (Table 3).

**Table 2:**
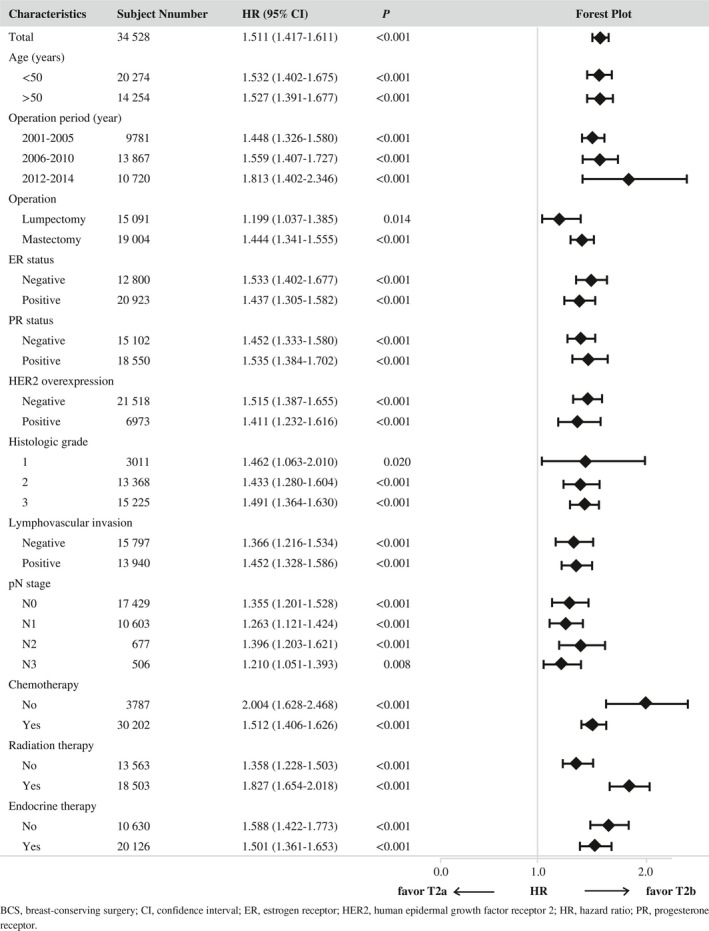
Hazard ratios regarding overall survival by subgroup analyses according to clinicopathologic risk factors

**Table 3:** Univariate and multivariate analyses regarding overall survival

Variable	Univariate analysis		Multivariate analysis[Link]	
	HR	95% CI	HR	95% CI
Tumor size				
T2a (>2.0 cm and ≤3.0 cm)	Reference		Reference	
T2b (>3.0 cm and ≤5.0 cm)	1.51	1.42‐1.61	1.26	1.18‐1.34
N category				
N0	Reference		Reference	
N1	1.70	1.58‐1.83	1.70	1.56‐1.85
N2	2.55	2.34‐2.79	2.73	2.47‐3.03
N3	5.11	4.69‐5.57	5.36	4.84‐5.94
Histologic grade				
1	Reference		Reference	
2	1.76	1.51‐2.06	1.40	1.19‐1.65
3	2.46	2.12‐2.87	1.69	1.43‐1.99
Lymphovascular invasion				
No	Reference		Reference	
Yes	1.83	1.70‐1.96	1.24	1.14‐1.34
ER status				
Negative	Reference		Reference	
Positive	0.61	0.57‐0.65	0.72	0.65‐0.79
PR status				
Negative	Reference		Reference	
Positive	0.58	0.55‐0.62	0.76	0.69‐0.83
HER2 overexpression				
Negative	Reference		Reference	
Positive	1.28	1.18‐1.38	0.965	0.89‐1.05
Chemotherapy				
No	Reference		Reference	
Yes	0.75	0.67‐0.83	0.51	0.45‐0.57
Radiation therapy				
No	Reference		Reference	
Yes	0.93	0.87‐1.00	0.75	0.69‐0.81
Endocrine therapy				
No	Reference		Reference	
Yes	0.66	0.62‐0.71	0.95	0.87‐1.05
Age				
≤50	Reference		Reference	
>50	1.51	1.43‐1.60	1.40	1.31‐1.50
Year of diagnosis				
2001‐2005	Reference		Reference	
2006‐2010	0.77	0.72‐0.82	0.78	0.72‐0.84
2011‐2014	0.61	0.53‐0.69	0.65	0.56‐0.75

CI, confidence interval; ER, estrogen receptor; HER2, human epidermal growth factor receptor 2; HR, hazard ratio; PR, progesterone receptor.

All significant risk factors for overall survival in univariate analyses except nuclear grade were incorporated into subsequent multivariate analysis using Cox's proportional hazard model. We excluded nuclear grade in our multivariate analysis because it was a potential confounder for histologic grade in its nature.

## 4 DISCUSSION

Cancer staging is central to the modern management of cancer patients. Any good staging system must be valid, reliable, and above all, it must be practical.^17^ A practical staging system must be suitable for daily use in a wide range of clinical environments and must not require diagnostic procedures that are not readily available to most practitioners. In this context, TNM staging system which was based on anatomic extent of malignant disease has been the most practical one for most of malignant diseases. Our attempt to subcategorize T2 in breast cancer was very practical, but holds a definite prognostic value. Although biologic features of breast tumor hold an evident clinical importance other than the current anatomic staging system, we do not need to abandon its practical value. Furthermore, new knowledge supporting a change in staging system might be created by facilitating clinical studies based on the new staging system itself.

The prognostic influence of T2 subcategorization in this study was independent of node status. Studies conducted over several decades have concluded that tumor size and the number of involved LN are positively correlated.^18^, ^19^, ^20^, ^21^ As T2 breast cancer size ranges from 2.0 to 5.0 cm, axillary LN status in T2 breast cancers might be more diverse than that in T1. Given that axillary node metastasis is one of the most powerful prognostic factors in breast cancer, we should also consider axillary node status when we assess the prognostic value of T2 subcategorization. In this study, patients with T2b tumors showed consistently worse outcomes in every node category than patients with T2a tumors (Figure 2). Furthermore, pathologic node category was a powerful prognostic factor in both T2 subcategories, although the distribution of node categories was quite different between the two T2 subcategories (Figure 3). These results suggest that tumor size is an independent prognostic factor within the range of T2 category regardless of axillary node status.

In this study, more than two‐thirds of 34 528 patients analyzed were subcategorized to T2a (Table 1). Characteristics of T2 category might be mainly determined by T2a subcategory being confined to the range of only 1 cm wide. Because patients with T2a tumors, as stated above, clearly showed better survival outcomes than patients with T2b tumors, this uneven distribution might have hidden the poor survival outcome of T2b subcategory representing relatively smaller proportion in T2 category. It is not reasonable that characteristics of T2 category in a whole are affected by those of T2a subcategory which represents only a small part of the whole range of T2 category.

Some subtypes of breast cancer might have enhanced propensity to metastasize when they are very small. On the other hand, some large breast tumors might metastasize late, if they metastasize at all.^6^, ^22^, ^23^, ^24^ Because of such heterogeneity, current anatomic staging of breast cancer may fail to accurately capture the behavior of some tumors or patient prognosis. This limitation of anatomic staging has resulted in the incorporation of ER status, HER2 status, grade, and molecular tumor characteristics into the 8th edition revision of AJCC cancer staging for breast cancer.^25^, ^26^ Unfortunately, the survival difference between the two T2 subcategories in this study was not fully explained by the model after adjusted for those biologic risk factors (Tables 2 and 3). Although the staging system starts to incorporate aspects of tumor biology, examining anatomic staging in more details might be still important.

The validity of T2 subcategorization in this study might be supported by several recent studies using SEER database. In the work of Wo et al,^27^ breast cancer‐specific mortality is positively correlated with tumor size in the range of T2 as well as T1. Although such correlation was pronounced in tumors less than 3.0 cm, it was unclear for tumors larger than 3 cm. The increase in breast cancer‐specific mortality reached a plateau despite increasing tumor size in tumors measuring 3 cm or more. Similar results have been reported in other recent studies based on SEER database.^9^, ^28^ Additionally, Lannin et al^29^ have reported that the distributions of biologic features of breast cancer categorized by ER, PR status, and histologic grade are different according to tumor size. In their study, favorable features (lower grade and hormone receptor positive) are more frequent but unfavorable features (higher grade and hormone receptor negative) are less frequent in smaller tumors. The distribution of biologic category showed marked difference according to tumor size in the range of 0.1‐3.0 cm. However, the difference was not evident when the tumor size was greater than 3.0 cm. Findings of these studies suggest that tumors larger than 3 cm might be different in their biologic features from tumors smaller than 3 cm. Thus, cutoff point of tumor size could be used to subdivide T2 category.

Our study has several limitations. First, data of adjuvant therapy were not complete in this analysis. As T2b breast cancers had relatively unfavorable biologic features, there might be differences in application of adjuvant therapy between the two groups. Within our dataset, adjuvant chemotherapy was more frequent among patients with T2b tumors, but adjuvant radiation and endocrine therapy were more common among patients with T2a tumors. These findings were not surprising considering BCS was much more frequent, and the proportion of ER‐positive disease was higher among T2a tumors than among T2b tumors. As our data of adjuvant therapy contain considerable missing values, unexpected deviation among them should be considered. However, this possible deviation might reduce survival difference taking the worse biologic features of T2b tumors into consideration. Thus, it is unlikely to introduce confounding effect pronouncing survival difference. In addition, tumor size was unknown in 15.9% of primary eligible 41 071 patients. This was inevitable because tumor size was not a required entry in KBCR, which could be omitted. However, there was no specific pattern of these omissions. Thus, they would be likely to be random. They are unlikely to change our result. Another limitation of the present study was in its study end point and relatively short follow‐up period. We defined OS as a study end point instead of disease‐specific survival (DSS) or disease recurrence (DR). OS is not ideal as an end point compared with DSS or DR. However, OS represents DSS even though it is less sensitive for the breast cancer‐specific mortality. For our purpose to define survival difference between the two T2 subcategories, OS would be used as a surrogate for DSS, because difference in OS is likely to be less prominent than that of DSS. Median follow‐up period for OS in this study was 65 months, which was short to detect late recurrences among ER‐positive breast cancers. As more than half of entire cohort with T2 tumors had ER‐positive breast cancers, further follow‐up for those population should be warranted. Finally, all patients enrolled in this study were Koreans (ethnically homogenous Far East Asian). The significance of T2 subcategorization and cutoff value in the current study might be limited to this ethnic group. However, given previous studies using SEER database, similar findings have been found in Western women.^9^, ^26^, ^27^

In conclusion, our study revealed that T2 category in breast cancer could be subcategorized into two subcategories: tumors ≤3 cm and tumors >3 cm. Those two subcategories clearly showed different OS after adjusting for known breast cancer prognostic factors. This result may reflect differences in biologic features between T2a and T2b. More detailed classification of tumor size might help us predict prognosis more accurately and provide tailored adjuvant therapy. The distinct prognostic difference between those T2 subcategories warrants further investigation.

## CONFLICT OF INTEREST

The authors declare that no actual or potential conflict of interest exists. The institutional review boards approved this study (Seoul Medical Center, 2017‐05‐001; Seoul National University Boramae Medical Center, 07‐2017‐9).

## Appendix 1

### 1.1

Sei Hyun Ahn (Asan Medical Center), Dong‐Young Noh (Seoul National University Hospital), Seok Jin Nam (Samsung Medical Center), Eun Sook Lee (National Cancer Center), Byeong‐Woo Park (Yonsei University Severance Hospital), Woo Chul Noh (Korea Cancer Center Hospital), Jung Han Yoon (Chonnam National University Hwasun Hospital), Soo Jung Lee (Yeungnam University Medical Center), Eun Kyu Lee (Seoul National University Bundang Hospital), Joon Jeong (Yonsei University Gangnam Severance Hospital), Sehwan Han (Ajou University School of Medicine), Ho Yong Park (Kyungpook National University Medical Center), Nam‐Sun Paik (Ewha Womans University Mokdong Hospital), Young Tae Bae (Pusan National University Hospital), Hyouk Jin Lee (Saegyaero Hospital), Heung kyu Park (Gachon University Gil Hospital), Seung Sang Ko (Dankook University Cheil Gen‐eral Hospital and Women's Healthcare Center), Woo‐Chan Park (The Catholic University of Korea Seoul St. Mary's Hospital), Young Jin Suh (The Catholic University of Korea St. Vincent's Hospital), Sung Hoo Jung (Chonbuk National University Hospital), Se Heon Cho (Dong‐A University Hospital), Sei Joong Kim (Inha University Hospital), Se Jeong Oh (The Catholic University of Korea Incheon St. Mary's Hospital), Byung Kyun Ko (Ulsan University Hospital), Ku Sang Kim (Ulsan City Hospital), Chanheun Park (Sungkyunkwan University Kangbuk Samsung Hospital), Byung Joo Song (The Catholic University of Korea Bucheon St. Mary's Hospital), Ki‐Tae Hwang (Seoul National University Boramae Medical Center), Je Ryong Kim (Chungnam National University Hospital), Jeoung Won Bae (Korea University Anam Hospital), Jeong‐Soo Kim (The Catholic University of Korea Uijeongbu St. Mary's Hospital), Sun Hee Kang (Keimyung University School of Medicine Dongsan Medical Center), Geumhee Gwak (Inje University Sanggye Paik Hospital), Jee Hyun Lee (Soonchunhyang University Seoul Hospital), Tae Hyun Kim (Inje University Busan Paik Hospital), Myungchul Chang (Dankook University Hospital), Sung Yong Kim (Soonchunhyang University College of Medicine Cheonan Hospital), Jung Sun Lee (Inje University College of Medicine Haeundae Paik Hospital), Jeong‐Yoon Song (Kyung Hee University Hospital at Gangdong), Hai Lin Park (Gangnam CHA University Hospital), Sun Young Min (Kyung Hee University Medical Center), Jung‐Hyun Yang (Konkuk University Medical Center), Sung Hwan Park (Catholic University of Daegu Hospital), Jong‐Min Baek (The Catholic University of Korea Yeouido St. Mary's Hospital), Lee Su Kim (Hallym University Hallym Sacred Heart Hospital), Dong Won Ryu (Kosin University Gospel Hospital), Kweon Cheon Kim (Chosun University Hospital), Min Sung Chung (Hanyang University Seoul Hospital), Hee Boong Park (Park Hee Boong Surgical Clinic), Cheol Wan Lim (Soonchunhyang University Bucheon Hospital), Un Jong Choi (Wonkwang University Hospital), Beom Seok Kwak (Dongguk University Ilsan Hospital), Young Sam Park (Presbyterian Medical Center), Hyuk Jai Shin (Myongji Hospital), Young Jin Choi (Chungbuk National University Hospital), Doyil Kim (MizMedi Hospital), Airi Han (Yonsei University Wonju Severance Christian Hospital), Jong Hyun Koh (Cheongju St. Mary's Hospital), Sangyong Choi (Gwangmyung Sung Ae Hospital), Daesung Yoon (Konyang University Hospital), Soo Youn Choi (Hallym University Kangdong Sacred Heart Hospital), Shin Hee Chul (ChungAng University Hospital), Jae Il Kim (Inje University Ilsan Paik Hospital), Jae Hyuck Choi (Jeju National University Hospital), Jin Woo Ryu (Chungmu Genral Hospital), Chang Dae Ko (Dr. Ko's breast Clinic), Il Kyun Lee (The Catholic Kwandong University International St. Mary's Hospital), Dong Seok Lee (Bun Hong Hospital), Seunghye Choi (The Catholic University of Korea St. Paul's Hospital), Youn Ki Min (Cheju Halla General Hospital), Young San Jeon (Goo Hospital), Eun‐Hwa Park (Ulsan University Gangneung Asan Hospital).
