# Supplementary material for: Clinical implication of subcategorizing T2 category into T2a and T2b in TNM staging of breast cancer
Source: Cancer Med. 2018 Oct 12;7(11):5514–24. doi: 10.1002/cam4.1831 (PMC6246943; doi:10.1002/cam4.1831)
Supplement: Supplementary file 1 [file CAM4-7-5514-s001.pdf]

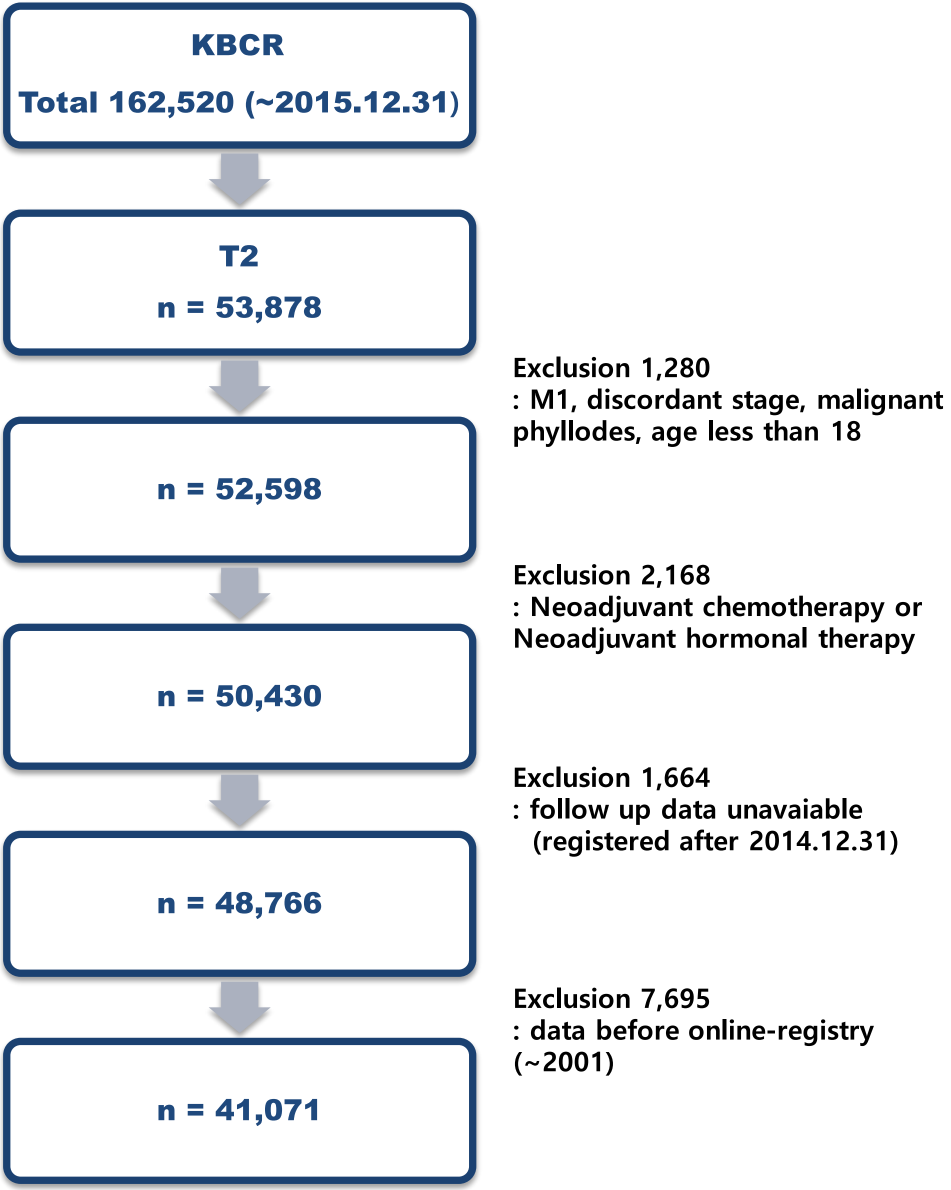
KBCR
Total 162,520 (~2015.12.31)
T2
n = 53,878
Exclusion 1,280
: M1, discordant stage, malignant phyllodes, age less than 18
n = 52,598
Exclusion 2,168
: Neoadjuvant chemotherapy or Neoadjuvant hormonal therapy
n = 50,430
Exclusion 1,664
: follow up data unavaiable (registered after 2014.12.31)
n = 48,766
Exclusion 7,695
: data before online-registry (~2001)
n = 41,071
