# Supplementary figures and images for: Clinical implication of subcategorizing T2 category into T2a and T2b in TNM staging of breast cancer
Source: Cancer Med. 2018 Oct 12;7(11):5514–24. doi: 10.1002/cam4.1831 (PMC6246943; doi:10.1002/cam4.1831)

**A**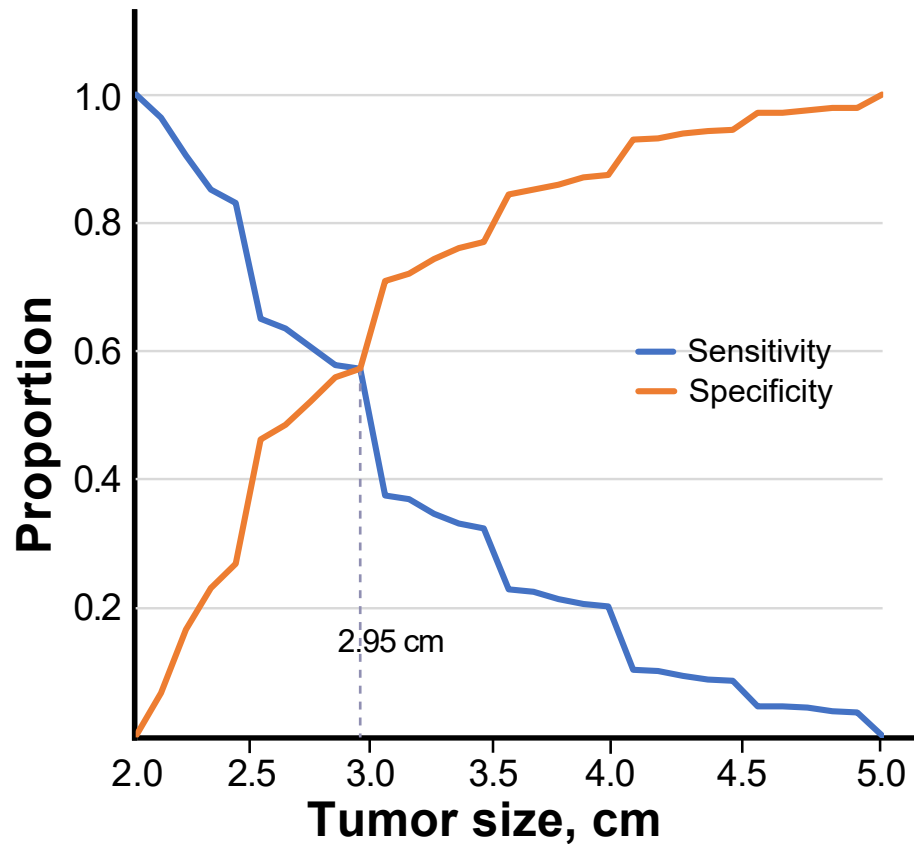**B**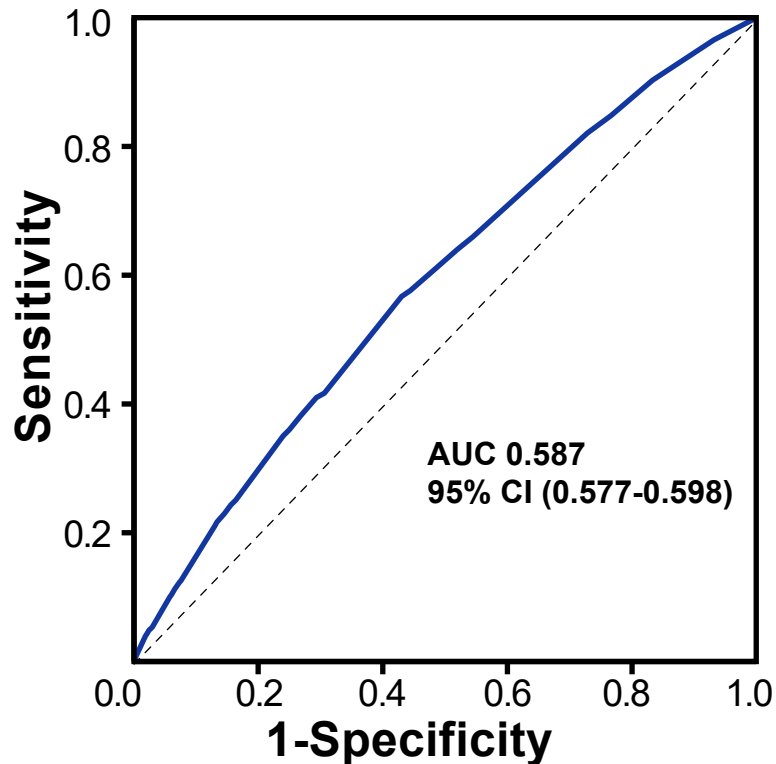

Supplement: Supplementary file 2 [file CAM4-7-5514-s002.pdf]
